# Epigenetic mechanisms of hypoxic preconditioning

**DOI:** 10.1186/2193-1801-4-S1-L39

**Published:** 2015-06-12

**Authors:** Elena Rybnikova, Mikhail Samoilov

**Affiliations:** Pavlov Institute of Physiology, Russian Academy of Sciences, Russian Federation, Russia

**Keywords:** Hypoxic preconditioning, brain hypoxic/ischemic tolerance, epigenetic mechanisms

Hypoxic preconditioning is a pre-exposure to the repetitive mild hypoxia which results in development of brain hypoxic/ischemic tolerance and cross-tolerance to injurious factors of another nature. The endogenous defense processes mobilized by hypoxic preconditioning and resulting in formation of brain tolerance are based on evolutionary acquired gene-determined mechanisms of neuroprotection and adaptation. Key event is an activation of pro-adaptive transcriptional factors HIF-1, CREB, NF-kB, c-Fos, NGFI-A and down-stream expression of their target genes in vulnerable brain neurons. An important role can thus be suggested for the epigenetic regulation of gene expression, in particular, acetylation of core nucleosome histones leading to changes in chromatin structure which ensure access of the transcriptional factors activated by the preconditioning to the promoters of target genes. It has been shown that hypoxic preconditioning considerably increases an acetylation status of all histones and, particularly, H3 in the neurons of rat hippocampus and neocortex, whereas injurious severe hypoxia causes global repression of histone acetylation. Diverse effects of the severe hypoxia and mild hypoxic preconditioning on the methylation of DNA and histones have also been observed. The complex of the epigenetic modifications induced by the hypoxic preconditioning is attributed to the relaxed DNA which becomes available for activation of gene expression by pro-adaptive transcriptional factors up-regulated by the preconditioning.

